# Clinical features of patients with fungal infections caused by CARD9 deficiency: a literature review of case reports

**DOI:** 10.3389/fcimb.2025.1615929

**Published:** 2025-07-16

**Authors:** Congchen Tang, Yalan Liu, Jiangchao Long, Xiaoju Lv

**Affiliations:** ^1^ Center for Infectious Diseases, West China Hospital, Sichuan University, Chengdu, Sichuan, China; ^2^ Intensive Care Unit, People’s Hospital of Dafang, Bijie, Guizhou, China

**Keywords:** CARD9 deficiency, fungal infection, gene mutation, clinical features, review

## Abstract

Caspase recruitment domain containing protein 9 (CARD9) deficiency is an autosomal-recessive primary immunodeficiency disorder, undermines the body’s capacity to combat fungal infections. In recent years, the number of reported cases of fungal infections associated with CARD9 deficiency has been increasing. This study undertook a systematic review of case reports, incorporating 89 patients with CARD9 deficiency complicated by fungal infections. The findings demonstrated that the patient population predominantly consisted of young and middle-aged individuals (33.43 ± 19.12 years, range: 1-91), and the majority (52 patients, 58.43%) developed the disease during childhood or adolescence. Significant geographical variations were observed in the distribution of gene mutations. Specifically, the c.820dupG mutation was predominantly found in East Asia, while the c.865C>T mutation was primarily found North Africa. Regarding the clinical manifestations, the most frequently affected sites were the skin, central nervous system, and lymph nodes, and the principal fungal pathogens identified were *Trichophyton* and *Candida*. Correlation analysis indicated that c.883C>T increased the likelihood of *Candida* infection (*p*=0.008, OR=10.421, 95% CI 1.849-58.748), c.865C>T increased the probability of *Trichophyton* infection (*p*=0.038, OR=5.760, 95% CI 1.098-30.217) and dematiaceous fungi infection (*p*=0.005, OR=9.653, 95% CI 2.019-46.153). According to the types of mutations, nonsense mutation increased the risk of dematiaceous fungi infection (p=0.014, OR=6.212, 95% CI 1.453-26.556). Notably, a proportion of patients succumbed to the disease, and this was predominantly associated with infections of the central nervous system, blood system, and viscera. This underscores the importance of adequate antifungal therapy and long-term follow-up for patients with CARD9 deficiency-related fungal infections.

## Introduction

CARD9 is a crucial adaptor protein in the innate immune response against fungal infections and its Online Mendelian Inheritance in Man (OMIM) number is 607212. Autosomal recessive CARD9 deficiency was first documented in 2009 within a consanguineous Iranian pedigree presenting with chronic mucocutaneous candidiasis (CMC) and dermatophytosis (Glocker et al., 2009). When the immune system detects fungal pathogens, CARD9 plays a pivotal role in the activated signaling pathways (Yazdi et al., 2023). Mutations in the CARD9 gene (NM_052813) result in CARD9 deficiency, which substantially compromises the body’s capacity to elicit an effective antifungal immune response. This disruption targets mechanisms primarily mediated by the C-type lectin receptor (CLR) and Toll-like receptor (TLR) families, which initiate defense responses against fungal pathogens (Drummond et al., 2018; Doron et al., 2021). In recent years, the number of reported cases of fungal infections associated with CARD9 deficiency has been gradually increasing. These infections present diverse clinical manifestations and can affect multiple organs and systems in the human body. Understanding the clinical features of patients with CARD9 deficiency-related fungal infections is of great significance for early diagnosis, appropriate treatment, and improving patient prognosis. However, due to the relatively rare study of CARD9 deficiency and the wide variety of fungal pathogens involved, the current comprehensive understanding of its clinical characteristics remains limited. Previous studies have been fragmented, and it is necessary to conduct a systematic review of case reports to summarize and analyze the existing data. This review aims to provide more perspectives by collecting and analyzing case reports from around the world. By systematically examining the clinical features, gene mutations, treatment strategies, and prognoses of patients with CARD9 deficiency-related fungal infections, we hope to provide valuable insights for clinicians and researchers in the fields of infectious diseases and immunology, facilitating better management of these complex cases.

## Materials and methods

### Literature search

The review process entailed a comprehensive exploration of all extant published literature on reported cases of fungal infections attributable to CARD9 deficiency. In the pursuit of relevant published works, a systematic search was conducted across the PubMed and China National Knowledge Infrastructure (CNKI) databases. The search terms employed were “CARD9”, “caspase recruitment domain deficiency” and “caspase recruitment domain containing protein 9”. Subsequently, the references of the initially selected papers underwent meticulous examination and screening. Articles of a review nature, those lacking detailed clinical data, and reports concerning patients without fungal infections were meticulously excluded from the analysis.

### Data extraction

The following data were extracted: publication year, first author, age of the patient at the time of reporting, age of onset of the patient, patient’s gender, site of infection, fungal culture results, mutation sites, treatment regimens, treatment outcomes, whether the patient died of the disease, and patient origin. According to Melanized Fungi in Human Disease (Revankar and Sutton, 2010), the dematiaceous fungi category was extracted. According to *Fungal Infection: Diagnosis and Management, Fourth Edition* (Fsbath, 2012), superficial fungal infections are defined as only infections confined to the outermost layers of the skin, nails, hair, and mucous membranes. Deep fungal infections include the subcutaneous mycoses and the systemic mycoses, defined as infections of the dermis, subcutaneous tissues, and adjacent bones, as well as infections involving internal organs and vital structures. Define invasive fungal infection according to the Consensus Definitions of Invasive Fungal Disease from the European Organization for Research and Treatment of Cancer and the Mycoses Study Group Education and Research Consortium (Donnelly et al., 2020). We distinguish the types of gene mutations through https://www.ncbi.nlm.nih.gov/clinvar.

Regarding the treatment outcomes, a subjective classification was employed, categorizing them into five distinct groups. The “not reported” category encompassed cases where treatment outcome information was unavailable. The “ineffective” category denoted cases in which, following systematic treatment, the patient’s general condition and the results of auxiliary examinations exhibited no signs of improvement. The “slightly improved” category referred to cases showing some degree of improvement, yet with a low likelihood of achieving complete clinical remission. The “partially improved” category applied to cases demonstrating improvement and a relatively high probability of attaining complete clinical remission. Finally, the “complete clinical remission” category signified cases where the patient’s fungal infection was eradicated, and organ functions were essentially restored.

### Statistical analysis

The data extracted from the study were analyzed by the SPSS 27.0 software. The Mantel-Haenszel test was used to analyze the association between different factors, with sex as the stratification factor. When the sample size (n) is≥40 and all the theoretical count under the null hypothesis (T) are≥5, choose the Pearson chi-square test. When n≥40 and at least one theoretical count meets 1≤T<5, use the continuity-corrected chi-square test (Yates’ correction). When n<40 or T<1, select Fisher’s exact test. To explore further correlations, univariate and multivariate binary logistic regression analysis were conducted. In the multivariate regression analysis, we included age, gender, and different pathogens to eliminate confounding. The outcomes of this analysis were presented in terms of odds ratios (ORs) and their corresponding 95% confidence intervals (CIs).

## Results

### Patient basic information

**Figure 1:**
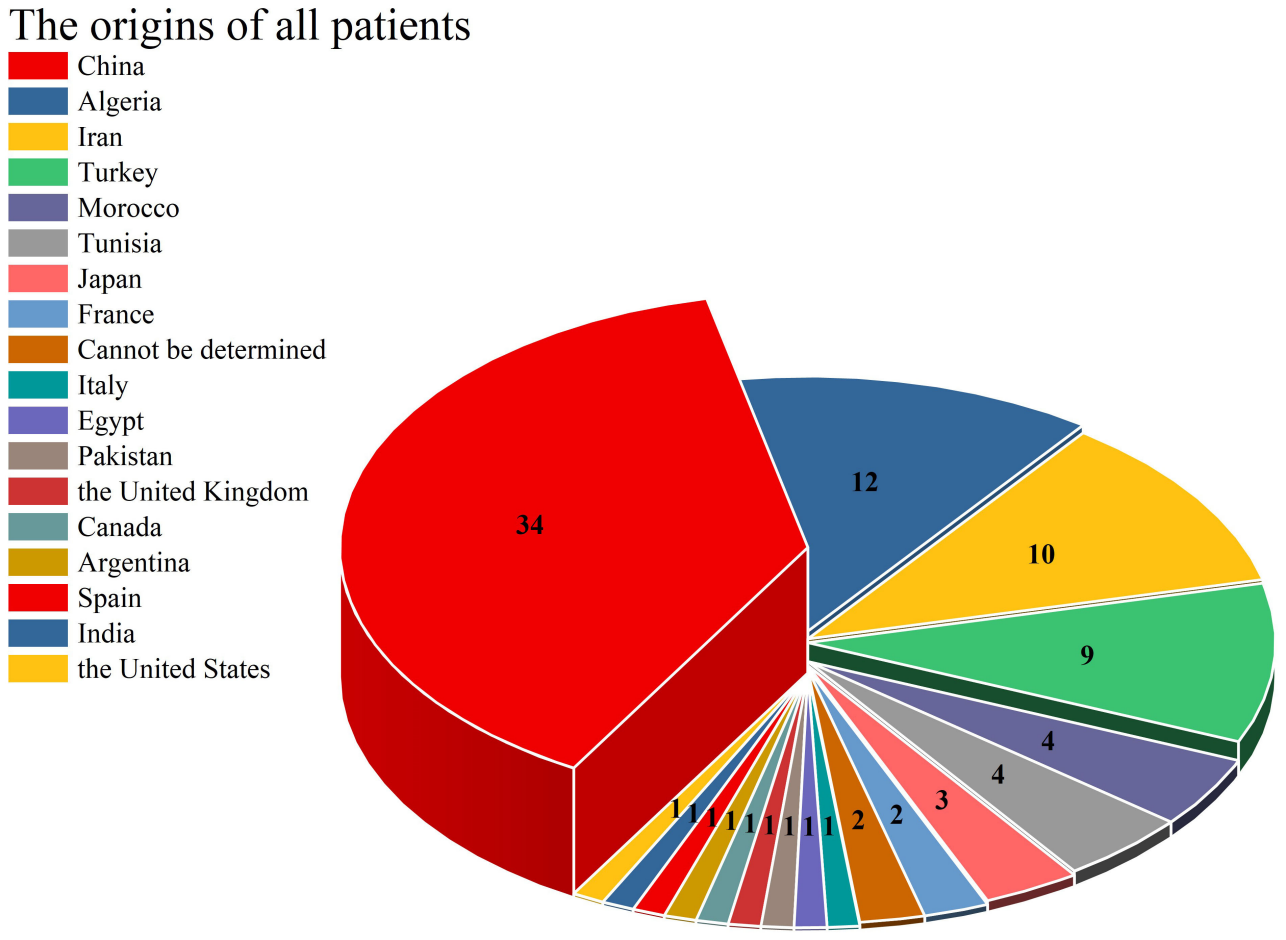
The origins of all patients.

In this study, a total of 58 articles were comprehensively incorporated, involving 89 patients with CARD9 deficiency, as detailed in **Table 1**. Among them, 48 patients were male (56.18%). The reported average age was 33.82 ± 18.90 years (range: 1-91), and 52 patients (58.43%) whose age of onset was less than 18 years old. The patients in this study originated from 17 distinct countries. As depicted in **Figure 1**, the countries with the highest 3 number of cases were China (34 cases, 38.20%), Algeria (12 cases, 13.48%), and Iran (10 cases, 11.24%).

**Table 1:** Statistical summary of the 82 enrolled patients’ information.

Patient	Kindreds	Reportd age	Onset age	Gender	Site of infection	Fungal culture results	Mutation site	Type of mutation	Other genetic mutation	Method of genetic testing	Treatment	Outcome	Death	Patient origin	References
P1	Kindred 1	19	3	Male	Oral cavity	*Candida*	Homozygous c.883C>T (p.Gln295Ter)	Nonsense	Not found	Sanger sequencing	KTCZ	Complete clinical remission	No	Iran	(Glocker et al., 2009)
P2	Kindred 1	–	<18	Male	Oral cavity, CNS	*Candida*	Homozygous c.883C>T (p.Gln295Ter)	Nonsense	Not found	Sanger sequencing	–	Ineffective	Yes	Iran	(Glocker et al., 2009)
P3	Kindred 1	50	42	Female	Skin, vagina	*Candida albicans*	Homozygous c.883C>T (p.Gln295Ter)	Nonsense	Not found	Sanger sequencing	–	–	No	Iran	(Glocker et al., 2009)
P4	Kindred 1	–	–	Female	Oral cavity, vagina, skin		Homozygous c.883C>T (p.Gln295Ter)	Nonsense	Not found	Sanger sequencing	–	–	No	Iran	(Glocker et al., 2009)
P5	Kindred 1	–	<18	Male	Skin	*-*	Homozygous c.883C>T (p.Gln295Ter)	Nonsense	Not found	Sanger sequencing	–	–	No	Iran	(Glocker et al., 2009)
P6	Kindred 1	–	<18	Female	Oral cavity, CNS	*-*	Homozygous c.883C>T (p.Gln295Ter)	Nonsense	Not found	Sanger sequencing	–	–	Yes	Iran	(Glocker et al., 2009)
P7	Kindred 1	–	<18	Female	Oral cavity, CNS	*Candida*	Homozygous c.883C>T (p.Gln295Ter)	Nonsense	Not found	Sanger sequencing	–	Ineffective	Yes	Iran	(Glocker et al., 2009)
P8	Kindred 2	75	6	Male	Skin, Scalp, Nails, Lymph nodes	*Trichophyton violaceum*	Homozygous c.865C>T (p.Gln289Ter)	Nonsense	Not found	Sanger sequencing	–	–	No	Algeria	(Lanternier et al., 2013)
P9	Kindred 2	29	2	Male	Skin, Scalp, Nails, Lymph nodes, CNS	*Trichophyton violaceum*	–	–	Not found	Sanger sequencing	GF+KTCZ+ITZ	Ineffective	Yes	Algeria	(Lanternier et al., 2013)
P10	–	40	9	Female	Skin, Scalp, Nails, Lymph nodes	*Trichophyton rubrum*	Homozygous c.865C>T (p.Gln289Ter)	Nonsense	Not found	Sanger sequencing	–	–	No	Algeria	(Lanternier et al., 2013)
P11	Kindred 3	56	8	Male	Skin, Scalp, Nails	*Trichophyton violaceum*	Homozygous c.865C>T (p.Gln289Ter)	Nonsense	Not found	Sanger sequencing	–	–	No	Algeria	(Lanternier et al., 2013)
P12	Kindred 3	34	8	Male	Skin, Scalp, Nails, Lymph nodes	*Trichophyton violaceum*	–	–	Not found	Sanger sequencing	–	–	Yes	Algeria	(Lanternier et al., 2013)
P13	Kindred 3	41	8	Female	Nails	*Trichophyton violaceum*	Homozygous c.865C>T (p.Gln289Ter)	Nonsense	Not found	Sanger sequencing	–	–	No	Algeria	(Lanternier et al., 2013)
P14	Kindred 4	43	19	Male	Skin, Scalp, Nails, Lymph nodes	*-*	Homozygous c.865C>T (p.Gln289Ter)	Nonsense	Not found	Sanger sequencing	–	–	No	Algeria	(Lanternier et al., 2013)
P15	Kindred 4	40	21	Male	Skin, Perineum, Scalp, Lymph nodes	*-*	Homozygous c.865C>T (p.Gln289Ter)	Nonsense	Not found	Sanger sequencing	–	–	No	Algeria	(Lanternier et al., 2013)
P16	Kindred 4	28	–	Male	Skin, Scalp	*-*	–	–	Not found	Sanger sequencing	–	–	Yes	Algeria	(Lanternier et al., 2013)
P17	Kindred 5	39	–	Male	Skin, Scalp, Lymph nodes	*Trichophyton violaceum*	Homozygous c.865C>T (p.Gln289Ter)	Nonsense	Not found	Sanger sequencing	GF+KTCZ	Partially improved	Yes	Algeria	(Lanternier et al., 2013)
P18	Kindred 5	37	–	Female	Nails, Skin	*-*	Homozygous c.865C>T (p.Gln289Ter)	Nonsense	Not found	Sanger sequencing	–	–	No	Algeria	(Lanternier et al., 2013)
P19	Kindred 6	40	–	Male	Skin, Bone, Lymph nodes	*Trichophyton rubrum*	Homozygous c.301C>T (p.Arg101Cys)	Missense	Not found	Sanger sequencing	–	–	No	Morocco	(Lanternier et al., 2013)
P20	Kindred 6	49	–	Female	Scalp, Nails	*-*	Homozygous c.301C>T (p.Arg101Cys)	Missense	Not found	Sanger sequencing	–	–	Yes	Morocco	(Lanternier et al., 2013)
P21	Kindred 7	91	6	Male	Skin, Scalp, Nails	*-*	Homozygous c.865C>T (p.Gln289Ter)	Nonsense	Not found	Sanger sequencing	–	–	No	Tunisia	(Lanternier et al., 2013)
P22	Kindred 7	44	12	Male	Scalp, Nails	*Trichophyton rubrum*	Homozygous c.865C>T (p.Gln289Ter)	Nonsense	Not found	Sanger sequencing	–	–	No	Tunisia	(Lanternier et al., 2013)
P23	Kindred 7	52	5	Female	Skin, Scalp, Nails, Lymph nodes	*Trichophyton rubrum and Trichophyton violaceum*	Homozygous c.865C>T (p.Gln289Ter)	Nonsense	Not found	Sanger sequencing	–	–	No	Tunisia	(Lanternier et al., 2013)
P24	–	62	6	Male	Skin, Scalp, Nails, Lymph nodes	*Trichophyton rubrum and Trichophyton violaceum*	Homozygous c.865C>T (p.Gln289Ter)	Nonsense	Not found	Sanger sequencing	–	–	No	Tunisia	(Lanternier et al., 2013)
P25	–	41	30	Male	CNS	*Candida albicans*	Homozygous c.271T>C (p.Tyr91His)	Missense	Not found	Whole exome sequencing	GM-CSF+VRC	Complete clinical remission	No	France	(Gavino et al., 2014)
P26	–	21	13	Male	Skin	*-*	Compound c.191_192insTGCT (p. Leu64fsTer59) and c.472C>T (p.Gln158Ter)	Frameshift and nonsense	Not found	Whole exome sequencing	ITZ+AMB	Ineffective	No	China	(Wang et al., 2014)
P27	–	17	6	Male	Skin	*-*	Homozygous c.819_820insG(p.Asp274fsTer60)	Frameshift	Not found	Whole exome sequencing	ITZ+AMB	Partially improved, relapse after discontinuation of the drug	No	China	(Wang et al., 2014)
P28	–	43	20	Female	Skin	*-*	Homozygous c.819_820insG(p.Asp274fsTer60)	Frameshift	Not found	Sanger sequencing	Surgical operation+ITZ	Partially improved	No	China	(Wang et al., 2014)
P29	–	64	48	Male	Skin	*-*	Homozygous c.819_820insG(p.Asp274fsTer60)	Frameshift	Not found	Sanger sequencing	ITZ+TBF	Partially improved	No	China	(Wang et al., 2014)
P30	–	24	3	Male	Skin, Oral cavity, Scalp, Nails	*Trichophyton mentagrophytes*	Homozygous c.302G>T (p. Arg101Leu)	Missense	Not found	Sanger sequencing	KTZ, ITZ, TBF, AMB	Slightly improved	No	Italy	Anete2015 (Grumach et al., 2015)
P31	–	4	1.5	Female	CNS	*Candida albicans*	Homozygous c.883C>T (p.Gln295Ter)	Nonsense	Not found	Sanger sequencing	AMB+5-FC+VRC followed by long-term FCZ	Complete clinical remission	No	Turkey	(Herbst et al., 2015)
P32	–	40	13	Male	Skin	*Trichophyton rubrum*	Homozygous c.865C>T (p.Gln289Ter)	Nonsense	Not found	Sanger sequencing	POS	Complete clinical remission	No	Egypt	(Jachiet et al., 2015)
P33	–	8	5	Female	CNS, Liver,	*Exophiala dermatitidis*	Homozygous c.52C>T (p. Arg18Trp)	Missense	Not found	Sanger sequencing	AMB+VRC	Ineffective	–	France	(Lanternier et al., 2015a)
P34	–	26	18	Female	Bone, Skin, Lung	*Exophiala* sp*inifera*	Homozygous c.967_969delGAG (p. Glu323de)	Deletion	Not found	Sanger sequencing	–	–	–	Iran	(Lanternier et al., 2015a)
P35	–	42	36	Female	CNS, Vagina,	*Candida albicans*	Homozygous c.208C>T (p. Arg70Trp)	Missense	Not found	Sanger sequencing	AMB +5-FC followed by long-term FCZ	Complete clinical remission	No	Turkey	(Lanternier et al., 2015b)
P36	–	7	7	Female	Skin, CNS, Oral cavity, Nails	*Candida albicans*	Homozygous c.208C>T (p. Arg70Trp)	Missense	Not found	Sanger sequencing	AMB +FCZ	Partially improved	No	Turkey	(Lanternier et al., 2015b)
P37	–	28	17	Male	Colon, Ileum, CNS,	*Candida glabrata*	Homozygous c.104G>A (p. Arg35Gln)	Missense	Not found	Sanger sequencing	FCZ, ITZ	Ineffective	No	Iran	(Lanternier et al., 2015b)
P38	–	37	34	Female	CNS, Oral cavity,	*Candida albicans*	Homozygous c.865C>T (p.Gln289Ter)	Nonsense	Not found	Sanger sequencing	AMB and 5-FC followed by long-term FCZ	Complete clinical remission	No	Morocco	(Lanternier et al., 2015b)
P39	–	34	26	Male	Oral cavity, Esophagus, Colon	*Candida albicans*	Homozygous c.883C>T (p.Gln295Ter)	Nonsense	Not found	Sanger sequencing	AMB+POS	Slightly improved	No	Pakistan	(Lanternier et al., 2015b)
P40	–	25	3	Male	CNS, Oral cavity, Skin	*Candida albicans*	Homozygous c.883C>T (p.Gln295Ter)	Nonsense	Not found	Targeted Resequencing	FCZ+AMB+CAS+G-CSF followed by long-term FCZ	Complete clinical remission	No	Turkey	(Celmeli et al., 2016)
P41	–	25	25	Female	Eye, Bone, Vagina	*Candida albicans*	Compound c.1138G>C (p. Ala380Pro) and c.951G>A (p.Arg317Arg)	Missense+ Silent	Not found	Whole exome sequencing	High-dose systemic antifungal agents followed by long-term KTZ	Partially improved	No	Britain	(Jones et al., 2016)
P42	–	45	9	Male	CNS, Oral cavity, Abdominal cavity, Liver, Lymph nodes	*Aspergillus, Candida.*	Homozygous c.883C>T (p.Gln295Ter)	Nonsense	SPAST mutation	Whole exome sequencing	Long-term KTZ	Complete clinical remission	No	Europe	(Rieber et al., 2016)
P43	–	12	12	Male	Blood vessel, Abdominal cavity, Skin	*Aspergillus fumigatus*	Homozygous c.3G>C (p. Met1Ile)	Missense	Not found	Targeted sequencing	Antifungal drug treatment +surgical operation+double umbilical cord stem cell transplantation	Ineffective	Yes	Africa	(Rieber et al., 2016)
P44	–	37	35	Female	Skin, Lymph nodes, Oral cavity	*Corynespora cassiicola*	Homozygous c.191_192InsTGCT(p. Leu64fsTer59)	Frameshift	Not found	Whole exome sequencing	AMB	Slightly improved	No	China	(Yan et al., 2016)
P45	–	47	10	Female	Skin, Scalp, Lymph nodes, CNS	*Trichophyton rubrum*	Homozygous c.865C>T (p.Gln289Ter)	Nonsense	Not found	Sanger sequencing	Long-term ITZ	Complete clinical remission	No	Algeria	(Boudghene Stambouli et al., 2017)
P46	–	34	16	Female	Skin, Oral cavity, CNS	*Phialophora verrucosa*	Compound c.104>A (p. Arg35Gln)+c.241G>A (p. Glu81Lys)	Missense	Not found	Sanger sequencing	GM-CSF+ITZ+TBF	Slightly improved	No	China	(Zhang et al., 2017)
P47	–	17	7	Female	CNS, Lung, Oral cavity	*Candida albicans*	Compound c.883C>T (p.Gln295Ter)	Nonsense+ Missense	Heterozygote NLRP12 mutation (c.910C>T; p. His304Tyr)	Targeted sequencing	VRC+AMB	Ineffective	Yes	Turkey	(Cetinkaya et al., 2018)
P48	–	8	8	Female	Colon	*Prototheca zopfii*	Homozygous c.781delG (p. Val261fs).	Frameshift	Not found	Whole exome sequencing	AMB	Partially improved	No	Turkey	(Sari et al., 2018)
P49	–	58	43	Female	Eye, CNS	*Candida albicans*	Compound c.184G>A and c.288C>T	Intronic (splicing)	Not found	Sanger sequencing	Long-term VRC	Complete clinical remission	No	Canada	(Gavino et al., 2018)
P50	–	28	26	Male	Skin	*Phialophora americana*	Homozygous c.819_820insG(p.Asp274fsTer60)	Frameshift	Not found	Sanger sequencing	ITZ+TBF	–	No	China	(Huang et al., 2019)
P51	–	24	12	Male	Skin, Esophagus, Bone	*Trichosporon asahii, Candida albicans*	Homozygous c.819_820insG(p.Asp274fsTer60)	Frameshift	Not found	Sanger sequencing	Long-term VRC	Complete clinical remission	No	China	(Quan et al., 2019)
P52	–	23	23	Male	CNS, Skin, Lymph nodes	*Exophiala dermatitidis*	Homozygous c.759dup (p. Lys254GlufsTer81)	Frameshift	Not found	Sanger sequencing	AMB +VRC	Ineffective	Yes	China	(Wang C. et al., 2019)
P53	–	35	17	Female	Skin, Lymph nodes, CNS	*Pallidocercospora crystallina*	Homozygous c.1118G>C (p. Arg373Pro)	Missense	Not found	Whole exome sequencing	ITZ+TBF+ surgical operation	Complete clinical remission	No	China	(Guo et al., 2019)
P54	–	7	5	Female	Oral cavity, Nails, CNS	*Candida albicans*	Homozygous c.208C>T (p. Arg70Trp)	Missense	Not found	Sanger sequencing	AMB + long-term FCZ	Complete clinical remission	No	Turkey	(Martin et al., 2019)
P55	–	46	46	Female	Skin	*Mucor irregularis*	Compound c.692C>T (p. p.Ser231Phe) and c.905_907delTCT (p.Ser302del)	Missense+ Frameshift	Not found	Sanger sequencing	AMB + long-term ITZ	Complete clinical remission	No	China	(Wang X. et al., 2019)
P56	–	27	16	Female	Skin	*Microsporum ferrugineum*	Compound c.883C>T (p.Gln295Ter) and c.1118G>C(p.Arg373Pro)	Nonsense+ Missense	Not found	Sanger sequencing	ITZ+TBF	Partially improved	No	China	(Zhang et al., 2019)
P57	–	10	9	Male	CNS, Oral cavity, Liver	*Candida albicans*	Homozygous c.819_820insG (p.Asp274fsTer60)	Frameshift	Not found	Whole exome sequencing	G–CSF+FCZ+5-FC	Complete clinical remission	No	China	(Du et al., 2020)
P58	–	12	9	Male	Colon, Esophagus, Oral cavity	*Histoplasma capsulatum*	Compound c.1204_1205insC (p. Cys402SerfsTer2) and c.1118G>C (p.Arg373Pro)	Frameshift+ Missense	Not found	Targeted sequencing	AMB followed by ITZ	Complete clinical remission	No	China	(Gao et al., 2020)
P59	–	31	16	Male	Skin, Nails, Lymph nodes	*Trichophyton rubrum, Trichophyton violaceum*, *Aspergillus fumigatus, and Aspergillus flavus.*	Compound c.271T>C (p.Tyr91His) and c.1269 + 18G>A	Missense+ Intronic	STS gene (Xp22.3)	Targeted sequencing	G-CSF+GM-CSF+ multiple antifungal drugs	Slightly improved, recurrent episodes	No	The United States of America	(Nazarian et al., 2020)
P60	–	56	32	Female	Skin, Lymph nodes, Lung	*Aspergillus nomius, Exophiala* sp*inifera*	Homozygous c.865C>T (p.Gln289Ter)	Nonsense	Not found	Sanger sequencing	Recombinant interferon γ-1b+ multiple antifungal drugs	Ineffective	Yes	Argentina	(Perez et al., 2020)
P61	–	48	17	Male	Skin	*Trichophyton rubrum, Candida albicans, Mucor irregularis*	Compound c.184 + 5G>T and c.951G>A (p.Arg317Arg)	Intronic (Splice)	Not found	Whole exome sequencing	ITZ+TBF	Complete clinical remission	No	China	(Wang X. et al., 2020)
P62	–	55	30	Female	Skin	*Phialophora expanda*	Homozygous c.819_820insG (p.Asp274fsTer60)	Frameshift	Not found	–	AMB+ITZ	Complete clinical remission	No	China	(Huang et al., 2020)
P63	–	<1	<1	Male	Lung, Liver, Skin, Spleen, Lymph nodes	*Talaromyces marneffei*	Compound c.1118G>C (p. Arg373pro) and c.610C>T (p.Asp204Asp)	Missense+ Silent	Not found	Whole exome sequencing	VRC	Complete clinical remission	No	China	(Ba et al., 2021)
P64	–	32	27	Male	Skin, Nails, Scalp, Lymph nodes	*Trichophyton rubrum*	Homozygous c.865C>T (p.Gln289Ter)	Nonsense	Not found	–	ITZ	Partially improved, relapse after discontinuation of the drug	No	Spain	(Benmehidi et al., 2021)
P65	–	4	4	Female	CNS, Spleen, Lymph nodes	*Exophiala dermatitidis*	Compound c.586A>G (p. Lys196Glu) and c.1118G>C (p.Arg373Pro)	Missense+ Missense	Not found	Targeted sequencing	AMB+VRC followed by TBF	Complete clinical remission	No	Japan	(Imanaka et al., 2021)
P66	–	26	17	Female	Skin	*Exserohilum rostratum*	c.1108C>T (p.Gln370Ter)	Nonsense	Not found	Targeted sequencing	ITZ+5-FC	Complete clinical remission	No	India	(Kalantri et al., 2021)
P67	–	37	<18	Male	CNS, Skin, Oral cavity	*Candida albicans*	Homozygous c.883C>T (p.Gln295Ter)	Nonsense	Not found	Sanger sequencing	Multiple antifungal drugs	Ineffective	Yes	Turkey	(Kuruoğlu et al., 2021)
P68	–	6	6	Male	CNS	*Alternaria*	Compound c. 1526G>A (p.Arg509Lys) and c.586A>G (p.Lys196Glu)	Missense+ Missense	Not found	Whole exome sequencing	Surgical operation+ VRC+ AMB followed by long term VRC	Complete clinical remission	No	China	(Lai et al., 2021)
P69	–	5	5	Male	Lung, Liver, Spleen, Abdominal cavity, Bone marrow,	*Talaromyces marneffei*	Compound c.440T>C(p.Leu147Pro) and c.586A>G(p.Lys196Glu)	Missense+ Missense	Not found	Medical Exome Sequencing	AMB+VRC	Ineffective	Yes	China	(You et al., 2021)
P70	–	55	23	Female	Skin	*Phialophora*	Homozygous c.819_820insG (p.Asp274fsTer60)	Frameshift	Not found	Exome Sequencing	AMB+ITZ	Partially improved	No	China	(Huang et al., 2022a)
P71	–	30	25	Male	Skin, Liver	*Trichosporon asahii*	Homozygous c.819_820insG (p.Asp274fsTer60)	Frameshift	Not found	–	VRC	Partially improved	No	China	(Huang et al., 2022b)
P72	–	28	23	Female	Skin, Nasal cavity, CNS	*Alternaria infectoria*	Homozygous c.865C>T (p.Gln289Ter)	Nonsense	Not found	Candidate Gene Sequencing	AMB+ITZ	Complete clinical remission	No	Turkey	(Paccoud et al., 2022)
P73	–	38	28	Male	Skin	*Trichophyton tonsurans*	Heterozygote c.596A>R (p. Lys196Glu)	Missense	Not found	Sanger sequencing	POS	Complete clinical remission	No	China	(Tan et al., 2022)
P74	–	68	67	Male	Skin, Lung,	*Corynespora cassiicola, Cladosporium*	Compound c.106C>T (p.Gln36Ter) and c.1118G>C (p.Arg373Pro)	Missense+ Missense	Not found	Whole exome sequencing	VRC	Complete clinical remission	No	China	(Wang et al., 2022)
P75	–	6	5	Male	Lung, Spleen, Lymph nodes, Rectum, Colon, Bone marrow	*Talaromyces marneffei*	Heterozygote c.820dupG (p. Asp274Ter)	Frameshift	CD40LG mutation (c.346G>A)	Whole exome sequencing	VRC+AMB	Slightly improved	No	China	(Yan et al., 2022)
P76	–	21	20	Female	Urethra	*Candida glabrata*	c.808-11G>I	Intronic	Not found	Whole exome sequencing	VRC, MFG, CAS	Complete clinical remission, relapse after discontinuation of the drug	No	China	(Deng et al., 2023)
P77	–	23	<18	Male	Skin, Lymph nodes, Parotid gland	*Trichophyton rubrum, Microsporum canis*	–	–	Not found	–	Surgical operation+ GF	Complete clinical remission	No	Morocco	(El Maati et al., 2023)
P78	–	14	12	Female	Lung	*Aspergillus terreus*	Homozygous c.86G>A (p. Arg29His)	Missense	Not found	Whole exome sequencing	Long term VRC	Complete clinical remission	No	Iran	(Fallahi et al., 2023)
P79	–	17	16	Male	Skin, CNS	*Prototheca wickerhamii*	c.820dupG (p. Asp274fs)	Frameshift	Not found	–	VRC+AMB	Partially improved	No	China	(Feng et al., 2023)
P80	–	40	40	Female	Skin, Lymph nodes	*Purpureocillium lilacinum*	Homozygous c.820dupG(p.Asp274fs)	Frameshift	Not found	–	VRC	Complete clinical remission	No	Japan	(Majima et al., 2023)
P81	–	12	12	Male	CNS, Oral cavity	*Candida albicans*	Compound c.1118G>C (p.Arg373Pro) and c.951G>A (p.Arg317Arg)	Missense+ Silent	Not found	Whole exome sequencing	AMB+VRC+5-FC followed by VRC+5-FC	Complete clinical remission	No	China	(Wang et al., 2023)
P82	–	29	25	Male	Skin	*Phialophora verrucosa*	Compound c.1118G>C (p.Arg373Pro) and c.820_821insG (p.Asp274fsTer60)	Missense+ Frameshift	Not found	Sanger sequencing	POS	Partially improved, relapse after discontinuation of the drug	No	China	(Zhang L. et al., 2023)
P83	–	66	59	Female	Skin	*Fusarium solaniae, Mucor irregularis*	Homozygous c.491delT	Frameshift	Not found	Whole exome sequencing	AMB	Complete clinical remission	No	China	(Zhou et al., 2023)
P84	–	21	13	Female	Skin, Lung	*Trichosporon asahii*	Homozygous c.820dupG (p. Asp274fs)	Frameshift	Not found	Exome Sequencing	VRC followed by ITZ	–	–	China	(Chen et al., 2023)
P85	–	41	16	Female	Skin	*Fusarium verticillioides*	Homozygous c.819_820insG (p.Asp274fsTer60)	Frameshift	Not found	Whole exome sequencing	ITZ	Complete clinical remission	No	China	(Zhang W. et al., 2023)
P86	–	80	77	Male	Skin, Lymph nodes	*Trichophyton rubrum*	Homozygous c.586A>G (p. Lys196Glu)	Missense	Not found	Whole exome sequencing	Surgical operation+ long term ITZ	Complete clinical remission	No	Japan	(Ansai et al., 2024)
P87	–	63	63	Male	Blood, Abdominal cavity	*T. marneffei*	c.35G>A (p.Ser12Asn)	Missense	Not found	Whole exome sequencing	CAS+VRC+AMB	Ineffective	Yes	China	(Liang et al., 2024)
P88	–	6	2	Female	CNS	*Exophiala dermatitidis*	Homozygous c.820dupG (p. D274GfsX60)	Frameshift	Not found	Whole exome sequencing	VRC+5-FC+AMB	Ineffective	Yes	China	(Ma et al., 2024)
P89	–	24	24	Female	CNS	*Candida albicans*	Homozygous c.184 + 5G>T	Intronic	Not found	Whole exome sequencing	CAS followed by FCZ+5-FC	Complete clinical remission	No	China	(Zhou et al., 2024)

CNS, Central Nervous System; VRC, Voriconazole; ITZ, Itraconazole; AMB, Amphotericin B; TBF, Terbinafine; FCZ; POS, Posaconazole; CAS, Caspofungin; FCZ, Fluconazole;5-FC, 5 - Fluorocytosine; GF, Griseofulvin; MFG, Micafungin; KTCZ, Ketoconazole; G-CSF, Granulocyte Colony Stimulating Factor; GM-CSF, Granulocyte Macrophage Colony Stimulating Factor.

### Gene variation distribution

**Figure 2:**
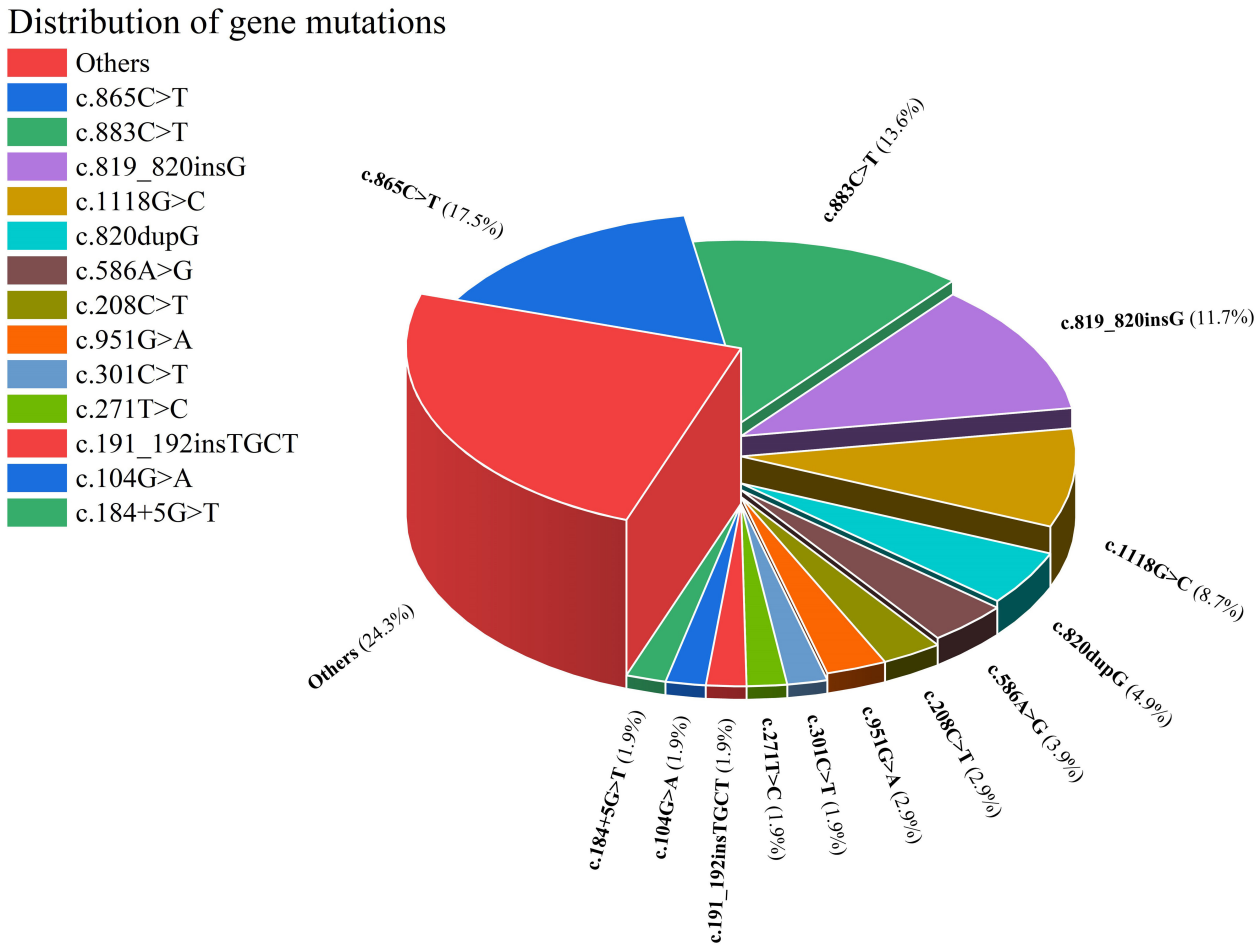
Distribution of gene mutations.

**Figure 3:**
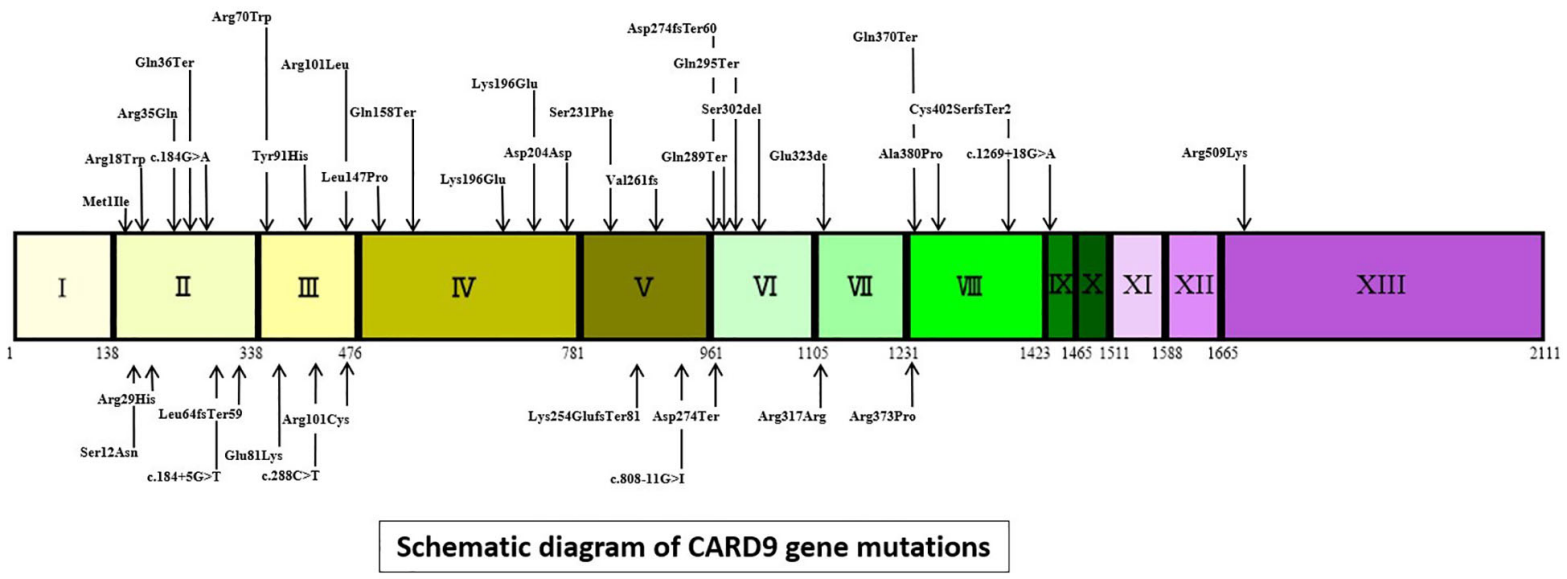
Schematic diagram of CARD9 gene mutations (intronic mutations represented by gene changes, other mutations denoted by amino acid changes. I to XIII = exons of CARD9, Coding DNA Sequence:155-1765).

As illustrated in **Figure 2**, this article comprehensively encompasses a total of 38 CARD9 gene mutations. The 5 most frequently occurring mutations are as follows: c.865C>T (18 cases), c.883C>T (14 cases), c.819-820insG (12 cases), c.1118G>C (9 cases) and c.820dupG (5 cases). The **“**others**”** segment in **Figure 2** encompasses 27 distinct gene mutations, each with a frequency of only one instance. These mutations are c.472C>T, c.302G>T, c.52C>T, c.967_969delGAG,c.1138G>C,c.3G>C,c.241G>A,c.781delG,c.184G>A,c.288C>T,c.759dup,c.692C>T,c.905_907delTCT,c.1204_1205insC,c.1269 **+** 18G>A,c.610C>T, c.1108C>T, c.1526G>A, c.440T>C, c.596A>R, c.106C>T, c.808-11G>I, c.86G>A, c.491delT, and c.35G>A. The CARD9 gene and related gene mutations are shown in **Figure 3**. There are 6 types of gene mutations: nonsense (30 cases), missense (29 cases), frameshift (23 cases), deletion (1 cases), silent (2 cases), and intronic (6 cases) mutation.

### Clinical features

**Figure 4:**
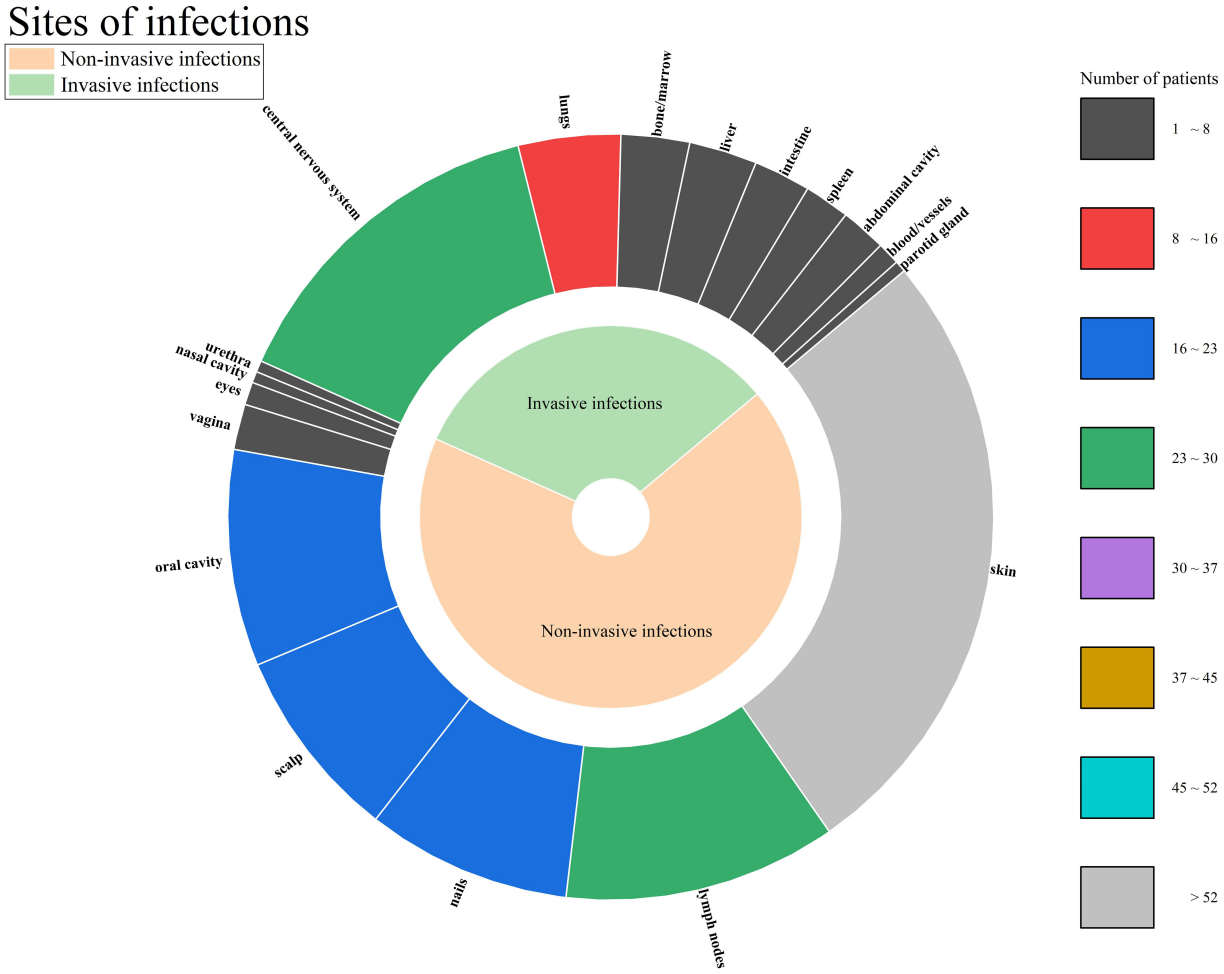
Site of infections.

**Figure 5:**
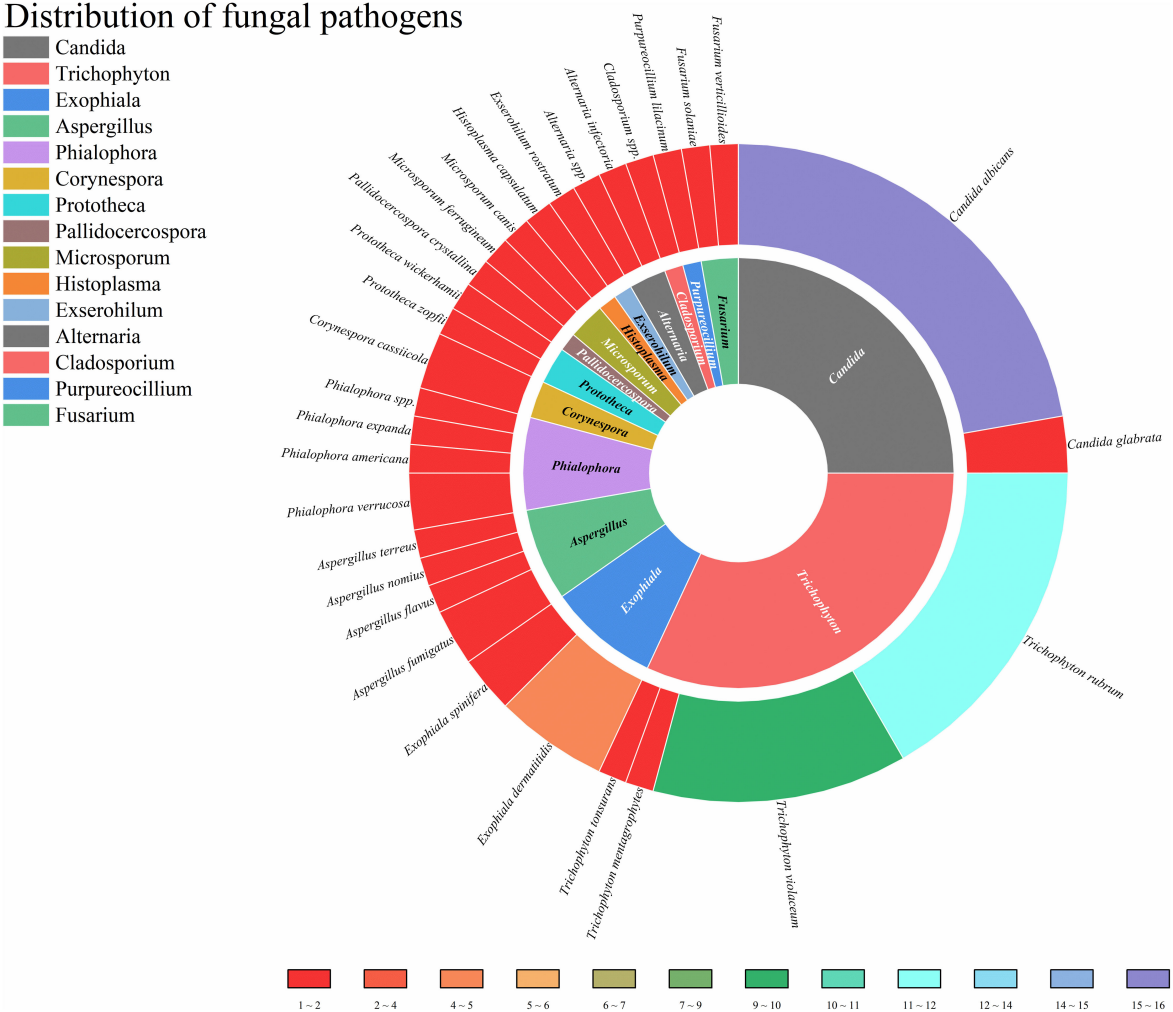
Distribution of fungal pathogens.

This study enrolled patients with fungal infections involving 18 distinct anatomical sites, as depicted in **Figure 4**. All patients had deep infections. Among them, 32.82% were invasive infections and 67.18% were non-invasive infections. The 3 most commonly affected sites were the skin, central nervous system, and lymph nodes. In terms of taxonomic classification at the genus level, *Trichophyton* and *Candida* were the 2 most prevalent pathogens, as illustrated in **Figure 5**. Dematiaceous fungi (16 cases) including: *Exophiala*, *Phialophora*, *Corynespora*, *Exserohilum*, *Alternaria*, and *Cladosporium*. In addition to standard antifungal pharmacotherapy, diverse treatment modalities were employed. Colony-stimulating factor (CSF) was administered to 5 patients (P18, P33, P39, P50, P52), surgical interventions were performed on 6 patients (P21, P36, P46, P61, P70, P79), and 1 patient (P53) received recombinant interferon γ-1b treatment. According to the clinical outcomes, they were classified into the following 5 categories: not reported (22 cases, 24.71%), ineffective (14 cases, 15.73%), slightly improved (6 cases, 6.74%), partially improved (13 cases, 14.61%), and complete clinical remission (34 cases,38.20%). Unfortunately, 16 patients (17.98%) succumbed to the disease.

### The relationship among genes, fungal pathogens and infection sites

To explore the relationships among various factors, we included the top 5 most frequent gene mutations (c.865C>T, c.819_820insG, c.1118G>C, c.883C>T, c.820dupG), gene mutations not in the top 5 (other mutations), *Trichophyton*, *Candida*, dematiaceous fungi, the top 3 most frequent anatomical sites (skin, CNS, lymph nodes), as well as invasive infections in the data analysis. Initially, the Mantel-Haenszel test was employed to assess the relationships between these factors. This statistical approach identified 18 significant associations, as detailed in **Table 2**: c.865C>T and *Trichophyton*, c.865C>T and dematiaceous fungi, c.865C>T and skin, c.865C>T and lymph nodes, c.865C>T and invasive infections, c.819_820insG and *Trichophyton*, c.819_820insG and lymph nodes, c.883C>T and *Candida*, other mutations and *Candida*, other mutations and skin, other mutations and central nervous system, other mutations and invasive infections, nonsense mutation and dematiaceous fungi, missense mutation and dematiaceous fungi, missense mutation and skin, missense mutation and invasive infections, frameshift mutation and *Trichophyton*, frameshift mutation and dematiaceous fungi. Subsequently, binary logistic regression analysis was carried out on these 19 identified associations to further quantify the relationships and estimate the strength of the associations, as presented in **Table 3**. The results indicated that c.883C>T increased the likelihood of *Candida* infections(p=0.008, OR=10.421, 95% CI 1.849-58.748), c.865C>T increased the probability of *Trichophyton* infections (p=0.038, OR=5.760, 95% CI 1.098-30.217) and dematiaceous fungi (p=0.005, OR=9.653, 95% CI 2.019-46.153). According to the types of mutation, nonsense mutation increased the risk of dematiaceous fungi infections (p=0.014, OR=6.212, 95% CI 1.453-26.556).

**Table 2:** The relationship between genes and infections.

Total patients (N=82)	*Trichophyton* (n=20)	*Candida* (n=18)	Dematiaceous fungi (n=16)	Skin (n=52)	Central nervous system (n=26)	Lymph nodes (n=24)	Invasive infection (n=44)
Site of mutation/P-value							
c.865C>T (n=18)	**<0.001** ^b^	0.114^b^	**<0.001** ^b^	**0.047** ^a^	0.121^a^	**0.006** ^a^	**0.002** ^a^
c.819_820insG (n=12)	**0.033** ^b^	0.919^b^	0.147^b^	0.220^b^	0.381^b^	**0.039** ^b^	0.126^a^
c.1118G>C (n=8)	0.208^b^	0.818^b^	0.319^b^	1.000^b^	1.000^b^	0.897^b^	0.368^b^
c.883C>T (n=14)	0.267^b^	**0.005** ^b^	0.388^b^	0.072^a^	0.068^b^	0.634^b^	0.167^b^
c.820dupG (n=5)	0.439^b^	0.505^b^	0.580^b^	0.520^b^	1.000^b^	0.970^b^	0.450^b^
Other Mutations (n=42)	0.739^a^	**0.042** ^a^	0.150^b^	**0.004** ^a^	**0.036** ^a^	0.222^a^	**0.027** ^b^
Type of mutation/P-value							
Nonsense(n=26)	0.059^a^	0.349^a^	**0.004** ^a^	0.692^a^	0.908^a^	0.299^a^	0.052^a^
Missense(n=29)	0.265^a^	0.362^a^	**0.033** ^a^	**0.015** ^a^	0.164^a^	0.207^a^	**0.012** ^a^
Frameshift(n=23)	**0.001** ^a^	0.070^a^	**0.013** ^b^	0.218^a^	0.082^a^	0.140^a^	0.248^b^
Deletion(n=1)	1.000^b^	1.000^b^	1.000^b^	1.000^b^	1.000^b^	1.000^b^	1.000^b^
Silent(n=2)	1.000^b^	0.067^b^	1.000^b^	0.253^b^	0.183^b^	0.893^b^	0.540^b^
Intronic(n=6)	0.763^b^	0.118^b^	0.580^b^	0.520^b^	1.000^b^	1.000^b^	0.866^b^

Bold represents having statistical differences.

The “n” in parentheses indicates the number of patients with a positive result for this item.

The superscripts on the right side of the P-value represent different test methods. “a” denotes the Pearson test, and “b” denotes the continuity-corrected test (Yates’ correction).

**Table 3:** The results of binary logistic regression analysis.

Project/Analysis	Univariate analysis		Multivariate analysis	
	P-value	OR (95%CI)	P-value	OR (95%CI)
**c.865C>T and *Trichophyton***	<0.001	7.636 (2.258-25.829)	0.038	5.760 (1.098-30.217)
**c.865C>T and dematiaceous fungi**	<0.001	18.543 (4.974-69.125)	0.005	9.653 (2.019-46.153)
c.865C>T and skin	0.998	–	–	–
c.865C>T and lymph nodes	0.008	4.464 (1.482-13.445)	0.412	–
c.865C>T and invasive infections	0.005	0.171 (0.051-0.581)	0.937	–
c.819_820insG and *Trichophyton*	0.998	–	–	–
c.819_820insG and lymph nodes	0.999	–	–	–
**c.883C>T and *Candida***	<0.001	8.585 (2.469-29.844)	0.008	10.421 (1.849-58.748)
Other mutations and *Candida*	0.018	0.309 (0.117-0.819)	0.131	–
Other mutations and skin	0.005	0.238 (0.088-0.643)	0.053	–
Other mutations and central nervous system	0.039	2.835 (1.054-7.627)	0.644	–
Other mutations and invasive infections	0.031	3.066 (1.109-8.475)	0.550	
**Nonsense mutation and dematiaceous fungi**	0.006	5.100 (1.584-16.422)	0.014	6.212 (1.453-26.556)
Missense mutation and dematiaceous fungi	0.047	0.206 (0.043-0.983)	0.103	–
Missense mutation and skin	0.015	0.303 (0.116-0.792)	0.059	0.304 (0.088-1.048)
Missense mutation and invasive infections	0.014	3.424 (1.286-9.113)	0.147	–
Frameshift mutation and *Trichophyton*	0.998	–	–	–
Frameshift mutation and dematiaceous fungi	0.998	–	–	–

**Bold** represents having statistical differences in Multivariate analysis.

## Discussion

CARD9, a pivotal downstream component of pattern recognition receptors (PRRs), plays a central role in mediating a cascade of inflammatory responses against invasive fungi, bacteria, viruses, and parasites. Mutations in the CARD9 gene, which lead to reduced expression and functional impairment, are associated with an autosomal recessive primary immunodeficiency disorder. This genetic defect renders affected individuals highly susceptible to microbial infections. The PRRs/Syk/CARD9 signaling pathway, situated downstream of PRRs, is one of the most well-characterized and fundamental signaling cascades in the immune response (Hu et al., 2022). CARD9-related C-type lectin receptors (CLRs) primarily include Dectin-1, Dectin-2, Dectin-3, and Mincle. Upon recognition of carbohydrate agonists, these CLRs recruit the tyrosine kinase Syk following Src kinase-mediated tyrosine phosphorylation of immunoreceptor tyrosine-based activation motif (ITAM)-like motifs (hem-ITAMs) or canonical ITAMs within their cytoplasmic tails (Rogers et al., 2005; Drummond et al., 2011). Syk serves as a pivotal signaling mediator, coupling activated immunoreceptors to downstream pathways in immune cells. Following recruitment, Syk undergoes phosphorylation, triggering the activation of protein kinase Cδ (PKCδ). This, in turn, facilitates the recruitment and phosphorylation of CARD9 at Thr231, initiating downstream signaling cascades (Wang Y. et al., 2020).Animals with a genetic deletion of Card9 are susceptible to challenge with a variety of fungal species, including *Candida albicans*, *Aspergillus fumigatus*, *Cryptococcus neoformans*, and some rarer dematiaceous fungi (Drummond et al., 2018).

The demographic profile of patients with CARD9-deficiency-associated fungal infections predominantly comprises young and middle-aged individuals. A significant proportion, specifically 57.32% (47 cases) of the patients, experience disease onset during childhood or adolescence. Notably, there are distinct geographical variations in the distribution of CARD9 gene mutations. For instance, the c.820dupG mutation is predominantly observed in East Asia, a finding that aligns with previous research by Tomomasa et al. (Tomomasa et al., 2024). Additionally, our study identified that the c.819-820insG and c.1118G>C mutations are uniquely present in the East Asian region, with 819-820insG being reported exclusively in China. In the case series presented by Lanternier et al. (Lanternier et al., 2013), all 12 patients with the c. 865C>T mutation were from Algeria, Morocco, and Tunisia. Over the past 12 years, 6 additional cases of this mutation have been reported, of which only 3 were from Spain, Turkey and Argentina, and the rest were from the above-mentioned North African countries, indicating that c.865C>T is mainly distributed in North Africa.

Fungal infections associated with CARD9 deficiency exhibit remarkable heterogeneity. The present study documented involvement of 18 distinct anatomical sites and identified 19 different genera of fungal pathogens. Among them, *Candida* and *Trichophyton* were the most isolated fungi. Meanwhile, fungal infections in CARD9-deficient patients showed a tendency toward severe invasiveness. According to the classification criteria of *Classification and Nomenclature of Fungi, Fungal diseases* (Fsbath, 2012), all patients met the criteria for deep infection (involving at least the dermis and subcutaneous tissues). According to the definition of invasive fungal infection (Donnelly et al., 2020), 32.82% of patients had definite invasive infections. Through correlation analysis, we found that the c.883C>T mutation significantly increased the likelihood of *Candida* infection, consistent with the analysis by Vaezi (Vaezi et al., 2018) and Dantas (Dantas et al., 2024). Moreover, the c.865C>T mutation was associated with an elevated probability of *Trichophyton* and dematiaceous fungi infection. A previous study (Vaezi et al., 2018) reported an association between c.819-820insG and disseminated phaeohyphomycosis (OR=2.42, 95%CI 1.84–3.2, p<0.001), and we did not find similar results.

The c.883C>T mutation in the CARD9 gene results from the substitution of cytosine (C) with thymine (T) at nucleotide position 883, leading to the premature formation of a stop codon. This reduces the short-term killing ability of CARD9-deficient neutrophils against unopsonized *Candida albicans* conidia (Gazendam et al., 2014; Corvilain et al., 2018). The c.865C>T mutation, where the cytosine (C) at nucleotide position 865 is replaced by thymine (T), results in a premature stop codon. This mutation inhibits the release of inflammatory cytokines such as IL-6, IL-1β, and IL-17A, potentially serving as the underlying mechanism for *Trichophyton* infections (Lanternier et al., 2013; Tan et al., 2022). This may explain the different pathogen susceptibilities associated with the two gene mutations. Dematiaceous fungi have been reported to cause subcutaneous and invasive infections, including chromoblastomycosis, phaeohyphomycosis, and mycetoma (McGinnis, 1983). A study investigating the response to pathogenic dematiaceous fungi in Card9-knockout mice found that the inability to control these fungi was associated with a lack of Th17 differentiation and reduced levels of tumor necrosis factor (TNF)-α, interleukin (IL)-1β, IL-6, and IL-17A in footpad homogenates (Wu et al., 2016). Previous research has not explored the relationship between mutation types and pathogens. We found that nonsense mutations increased the risk of dematiaceous fungi infections, yet the c.883C>T mutation, a relatively frequent nonsense mutation, did not exhibit this association. This discrepancy may be related to epidemiological differences. Although there is limited epidemiological data on dematiaceous fungi in Africa, a study on chromoblastomycosis prevalence, showed that Africa has the second-highest incidence after South America, while the c.883C>T mutation is absent in both regions.

Among the 82 patients included in this study, 13 succumbed to the disease. The majority of these fatal cases were associated with infections of the central nervous system, blood system, and/or viscera. This poor prognosis can be attributed, at least in part, to the reduced effectiveness to antifungal medications, which is a consequence of genetic defects in these patients. The prognosis of CARD9 patients is associated with co-existing mutations in other genes, some of which may exhibit synergistic effects. For example, co-mutations in the DOCK8 gene can lead to severe fungal infections (El Hawary et al., 2022). The genetic heterogeneity of inborn errors of immunity and diagnostic delays in atypical cases lead to significant morbidity and mortality. Establishing a definitive genetic diagnosis is crucial for patient management (Ripen et al., 2021). Among the patients included in this study, 28.05% (23/82) of the patients underwent whole exome sequencing. Only 4 cases were found to have mutations in other genes: P35 (SPAST mutation) (Rieber et al., 2016), P40 (NLRP12 mutation) (Cetinkaya et al., 2018), P52 (STS gene mutation) (Nazarian et al., 2020), and P68 (CD40LG mutation) (Yan et al., 2022). The latter 3 gene mutations are associated with infections, and in these 3 patients, the disease is more severe and the treatment is more difficult. Granulocyte colony stimulating factor (G-CSF) and granulocyte macrophage colony stimulating factor (GM-CSF) exert pleiotropic effects on the innate immune system by enhancing the function of human neutrophils (Lin et al., 2024). While their efficacy has been demonstrated in individual case reports (Gavino et al., 2014; Du et al., 2020), large-scale clinical trials are still lacking. Nevertheless, they represent valuable salvage treatment options for patients who do not respond adequately to conventional antifungal therapy.

In conclusion, CARD9 deficiency should be considered in the differential diagnosis of patients presenting with progressive fungal infections of unknown etiology. Early initiation of antifungal treatment is crucial for improving patient outcomes, and long-term prophylactic treatment and regular follow-up are essential components of comprehensive management strategies.

## Limitations

1. Our judgment of the patients’ clinical outcomes was subjective and only represented their conditions at that time, which might lead to a certain degree of bias.
2. There was no subjective classification of anatomical sites, such as the scalp and skin. However, for the integrity of the data, we directly extracted the sites stated in the articles. This might have some impact on the results.
3. Limited by the low prevalence of CARD9 deficiency, the statistical results may not reflect the true situation, especially for the interpretation of OR values.
4. This study did not include all CARD9 patients. It only included case reports and case series, and excluded patients without detailed clinical data and those with non-fungal infections.

## Conclusion

In the contemporary landscape of medical research, there has been a burgeoning focus on non-HIV-associated opportunistic infections, which has emerged as a crucial area of investigation due to their increasing prevalence and clinical significance. This study retrospectively analyzed 82 patients with CARD9 deficiency complicated by fungal infections and found significant differences in clinical symptoms, fungal pathogens, and gene mutation sites. It provides potential relationships between gene mutations, pathogens, infection sites, and regional distributions, aiming to enhance the understanding of this disease.
